# Machine Learning Model for Predicting Walking Ability in Lower Limb Amputees

**DOI:** 10.3390/jcm13226763

**Published:** 2024-11-10

**Authors:** Aleksandar Knezevic, Jovana Arsenovic, Enis Garipi, Nedeljko Platisa, Aleksandra Savic, Tijana Aleksandric, Dunja Popovic, Larisa Subic, Natasa Milenovic, Dusica Simic Panic, Slavko Budinski, Janko Pasternak, Vladimir Manojlovic, Milica Jeremic Knezevic, Mirna Kapetina Radovic, Zoran Jelicic

**Affiliations:** 1 Faculty of Medicine, University of Novi Sad, 21000 Novi Sad, Serbia; enis.garipi@mf.uns.ac.rs (E.G.); aleksandrasavic@uns.ac.rs (A.S.); tijana.aleksandric@mf.uns.ac.rs (T.A.); dunja.popovic@mf.uns.ac.rs (D.P.); larisa.subic@mf.uns.ac.rs (L.S.); natasa.milenovic@mf.uns.ac.rs (N.M.); dusica.simic-panic@mf.uns.ac.rs (D.S.P.); slavko.budinski@mf.uns.ac.rs (S.B.); janko.pasternak@mf.uns.ac.rs (J.P.); vladimir.manojlovic@mf.uns.ac.rs (V.M.); milica.jeremic-knezevic@mf.uns.ac.rs (M.J.K.); 2 Center for Physical Medicine and Rehabilitation, University Clinical Center of Vojvodina, 21000 Novi Sad, Serbia; nedeljko.platisa@kcv.rs; 3 Faculty of Technical Sciences, University of Novi Sad, 21000 Novi Sad, Serbia; arsenovic.jovana@uns.ac.rs (J.A.); mirna.kapetina@uns.ac.rs (M.K.R.); jelicic@uns.ac.rs (Z.J.); 4 Special Hospital for Rheumatic Diseases, 21000 Novi Sad, Serbia; 5 Clinic for Vascular and Endovascular Surgery, University Clinical Center of Vojvodina, 21000 Novi Sad, Serbia

**Keywords:** amputation, rehabilitation, recovery of function, support vector machines

## Abstract

**Background/Objectives**: The number of individuals with lower limb loss (LLL) is rising. Therefore, identifying the walking potential in individuals with LLL and prescribing adequate prosthetic systems are crucial. Various factors can influence participants’ walking ability, to different extents. The aim of the present study was to apply machine learning methods to develop a predictive mode. This model can assist rehabilitation and limb loss care teams in making informed decisions regarding prosthesis prescription and predicting walking ability in individuals with LLL. **Methods**: The present study was designed as a prospective cross-sectional study encompassing 104 consecutively recruited participants with LLL (average age 62.1 ± 10.9 years, 80 (76.9%) men) at the Medical Rehabilitation Clinic. Demographic, physical, psychological, and social status data of patients were collected at the beginning of the rehabilitation program. At the end of the treatment, K-level estimation of functional ability, a Timed Up and Go Test (TUG), and a Two-Minute Walking Test (TMWT) were performed. Support vector machines (SVM) were used to develop the prediction model. **Results**: Three decision trees were created, one for each output, as follows: K-level, TUG, and TMWT. For all three outputs, there were eight significant predictors (balance, body mass index, age, Beck depression inventory, amputation level, muscle strength of the residual extremity hip extensors, intact extremity (IE) plantar flexors, and IE hip extensors). For the K-level, the ninth predictor was The Multidimensional Scale of Perceived Social Support (MSPSS). **Conclusions**: Using the SVM model, we can predict the K-level, TUG, and TMWT with high accuracy. These clinical assessments could be incorporated into routine clinical practice to guide clinicians and inform patients of their potential level of ambulation.

## 1. Introduction

Predicting functional outcomes post-amputation is a significant challenge in the field of rehabilitation for individuals with lower limb loss (LLL). Prediction of walking ability and the use of prosthetics is primarily based on clinical expertise and personal opinions [1]. Furthermore, the number of individuals with LLL is rising. It is estimated that up to 2.3 million individuals in the U.S. were coping with limb loss in the year 2024 [2]. It is expected that this number will surpass 3.6 million in 2050 [3]. In Europe, the trends in lower extremity amputation incidence vary significantly across countries, with increasing trends in six out of nineteen countries between 1990 and 2017 [4]. To improve functionality and enhance the quality of life in people with LLL, it is crucial to prescribe and fit the prosthesis accurately [5]. For this purpose, it is of great importance to identify the potential for walking with prosthesis and prescribe adequate prosthetic components [6,7]. The K-level system, introduced in the U.S., is a grading scale used to assess a patient’s functional capabilities and determine the appropriate prosthetic components. It is primarily utilized for the prescription of prosthetic devices and functional assessment to ensure that the prosthesis meets the individual’s mobility needs. Health insurance companies in the U.S. often require K-level assessments to approve coverage for specific prosthetic components [8]. It is estimated that between 50% and 90% of individuals with LLL will be able to walk with a prosthesis [9,10,11]. However, it remains unclear which factors are the most critical predictors of the functional outcome [12].

In the context of LLL, a wide array of potential predictors for walking ability and functional outcomes has been identified in the literature. However, there is often contradictory evidence with mixed results regarding their predictive power, and many studies have focused on individual variables or smaller subsets. This gap could be addressed by selecting a comprehensive set of variables, including physical, psychological, and social factors. This approach would enable a more comprehensive assessment of the collective influence of key predictors on walking ability and functional outcomes in individuals with LLL.

For example, atherosclerosis, diabetes, or both, frequently seen in individuals with LLL, are associated with the reduction of physical and cognitive capacity [13]. Previous studies have suggested that factors such as the etiology of limb loss and tobacco-use may significantly influence walking ability in lower limb amputees, with tobacco-use exerting negative influence [13,14,15,16,17]. Higher amputation levels and older age are related to decreased functionality, as both factors adversely affect the functional outcome [7,13,15]. However, it is essential to note that older individuals often regain their walking ability with prostheses [18]. Emerging studies suggest a confounding effect of comorbidities and physical status—not age, per se—that impacts the functional outcome [19,20]. While a considerable number of studies indicate that age negatively influences walking capability, they also emphasize that age alone should not be a decisive factor in determining eligibility for prosthetic use [21]. Men undergo lower limb amputation more often than women; however, gender influence on functionality is not clear [16,22,23]. Sufficient muscle strength of the lower limbs is a precondition for successful ambulation with prosthesis. Indeed, muscle strength of the residual limb hip extensors was a significant predictor of walking distance in individuals with LLL [14]. Restriction in the range of motion of the residual limb could be a negative predictor of successful ambulation after lower limb amputation [14,16]. In addition to demographic and physical factors, the impact of social support should not be underestimated [15,24]. It encompasses emotional, informational, and instrumental assistance from family, friends, and healthcare providers. Studies have shown that strong social support can significantly improve mental health, adherence to rehabilitation protocols, and overall quality of life. It can also enhance motivation and provide a buffer against the psychological stress associated with LLL, ultimately leading to better functional outcomes [15,25].

While these factors have been individually explored in various studies, their combined effect, along with other physical, psychological, and social parameters, has not been thoroughly investigated. Our study seeks to bridge this gap by analyzing a comprehensive set of predictors.

Recent advancements in machine learning applications for lower limb amputees, as demonstrated by Quek et al. (2024) and Nsugbe et al. (2024), have shown promising results in predicting ambulatory capacity and quantifying mobility using both clinical and sensor-based data. By incorporating factors such as ethnicity, amputation level, and pre-amputation mobility, Quek et al. provided an early post-operative prognostic model, while Nsugbe et al.’s use of wearable sensors added valuable real-time mobility insights [26,27].

Methods from previous research have offered some insights, but may not have fully captured the complex interactions among the numerous variables that influence walking ability [28]. A potential solution is the introduction of more sophisticated data analysis, such as machine learning (ML). Recent strategies have emphasized a multifactorial approach to predicting functional outcomes, integrating both clinical assessments and machine learning techniques to enhance prediction accuracy [26,27].

ML has long had a significant impact on medical diagnosis and prognosis [29,30,31]. Recent developments in machine learning provide promising solutions for improving predictive accuracy and customizing rehabilitation strategies [26,27]. Developing a robust ML model that accurately predicts rehabilitation outcomes could substantially advance medical decision-making. Such a model could be integrated into decision support systems, aiding healthcare professionals in selecting the most appropriate prosthetics components and tailoring individualized rehabilitation treatments.

The goal of the present study is to create a robust prediction model utilizing a support vector machine (SVM) to improve the accuracy of predicting walking ability in individuals with LLL [32,33,34,35,36]. A decision tree was used to identify key physical, psychological, and social variables affecting walking ability [37,38,39,40,41].

## 2. Materials and Methods

### 2.1. Data Resources

This prospective cross-sectional study included 104 consecutively recruited individuals with LLL admitted to the Medical Rehabilitation Clinic of the Clinical Centre of Vojvodina (Novi Sad, Serbia) for prosthetic fitting and gait training between 2010 and 2013. The study was approved by the Ethical Board of the Clinical Centre of Vojvodina Novi Sad, Serbia) (No 00-02/367). All participants provided informed consent prior to enrollment in the study.

The inclusion criteria for this study were participants with unilateral transtibial or transfemoral amputation who were admitted to the clinic for the first time for prosthetic fitting and training.

Participants were excluded if they had previously undergone prosthetic fitting and gait training, had a bilateral amputation (except the contralateral toe amputation), or had incomplete medical documentation.

All participants underwent a rehabilitation program at the Medical Rehabilitation Clinic. The program averaged 26 ± 8 days and lasted approximately 2 h each session. It included exercises targeting balance, strength, and range of motion, prosthesis gait training, feedback training, proprioceptive exercises, and training in the use of assistive devices. The program was individualized and adjusted to each patient’s specific needs, and was provided by two licensed physiotherapists with over ten years of experience.

### 2.2. Collected Variables

The parameters were chosen to cover a broad spectrum of physical, psychological, and social factors relevant to walking ability and prosthesis prescription.

Data collection was conducted in two phases. The first phase was completed at the beginning of the rehabilitation treatment, during which general data about age, gender, and educational level (number of years of education) were collected. The number of comorbid conditions was assessed by the Functional Comorbidity Index (FCI) [42], with the score determined by the consensus of two independent physicians. In addition, the presence of diabetes mellitus (DM) and body mass index (BMI) (calculated from values of height and weight (prosthesis excluded), according to the formula “BMI = weight/(height)^2^ (kg/m^2^))” were noted as separate variables.

Participants were grouped by factors such as etiology of limb loss and tobacco-use based on evidence from the literature suggesting these as potential predictors of walking ability. According to the cause of amputation, participants were divided into three groups, as follows: peripheral vascular disease, traumatic causes, and amputations due to other causes. Information about the level of amputation (transfemoral vs. transtibial), the presence of phantom limb pain (PLP), and the time passed from the amputation to the moment of the data collection were also gathered. Participants were further categorized by their tobacco smoking habit into smokers, non-smokers (those who quit more than 6 months ago), and ex-smokers (those who quit less than 6 months ago).

Balance was tested on the intact extremity (IE). Participants were asked to maintain their balance while standing on the IE for as long as possible, and that time was recorded using a stopwatch. According to their ability to keep balance, they were grouped into the following four categories: balance on the IE not possible, possible with support, balance possible without support for less than 10 s, and balance possible without support for more than 10 s [15].

The residual limb’s extension range of motion in the hip (and knee in transtibial amputees) was measured using a goniometer. For the hip extension evaluation, participants were positioned lying on their sides on the IE. The stationary arm of the goniometer was perpendicular to a line drawn between the anterior superior iliac spine and the posterior superior iliac spine; the fulcrum was positioned over the greater trochanter; and the moving arm was aligned with the lateral epicondyle of the femur. The knee extension evaluation was performed in a supine position with the stationary arm aligned with the greater trochanter of the femur, the fulcrum positioned over the lateral epicondyle of the femur, and the moving arm of the goniometer aligned with the lateral malleolus [43]. The presence of flexion contractures in the residual limb was defined as a hip extension of less than 0° or a knee extension of less than −10° [44].

Muscle strength of the residual extremity (RE) hip extensors, IE plantar flexors, and IE hip extensors were performed by using body weight as resistance to the muscle activity. Therefore, hip extensors were tested in the supine position, according to Perry et al.’s recommendation [45]. Ankle plantar flexors were tested while subjects were standing on the IE. Muscle strength was graded from 0 (no trace of muscle contraction) to 5 (normal muscle strength) for IE, and from 0 (no trace of muscle contraction) to 4 (good muscle strength) for RE. Muscle strength evaluations were conducted by two experienced physical therapists, each with over ten years of experience working with individuals with lower limb loss. Both raters were trained and evaluated using the criteria proposed by Daniels and Worthingham to ensure consistency across all evaluations [46].

Experienced clinical psychologist N.P. (the co-author of the present study) evaluated cognitive function among participants at the beginning of the rehabilitation treatment using the Mini Mental State Exam (MMSE). The MMSE is a screening test designed to evaluate cognitive impairment in older adults, consisting of 11 items with a total score range from 0–30, where lower scores indicate a higher level of cognitive deterioration [47]. The MMSE has been used in previous studies for cognitive evaluation in individuals with LLL [48]. Depressive symptoms were assessed using the Beck depression inventory (BDI), which is scored from 0–63, with higher scores indicating a greater degree of depressive symptoms. The BDI has demonstrated good reliability, with Cronbach’s alpha values of 0.82 [49,50,51,52].

The Multidimensional Scale of Perceived Social Support (MSPSS) was employed to measure the perception of support from family, friends, and a significant other. The instrument consists of 12 items, rated on a 7-point Likert scale from 1 (very strongly disagree) to 7 (very strongly agree), with higher scores indicating better perceived support. The MSPSS generally shows good internal consistency, with Cronbach’s alpha values of 0.88 [50,53].

At the end of the rehabilitation treatment, the participants were classified according to their K-level [8]. K-levels are a widely used classification system designed to assess the level of mobility in individuals with lower limb loss, providing a basis for prosthesis prescription. However, K-level is widely used for functional assessment of individuals with LLL [54]. The system ranges from K0 to K4, defined as follows: K0 indicates that the individual does not have the ability or potential to ambulate or transfer safely with or without assistance; however, individuals at this level may still use a prosthesis for aesthetic purposes, which can improve their quality of life. K1 refers to those who have the ability to use a prosthesis for basic transfers or ambulation, making them limited, low-level ambulators. K2 describes individuals classified as community ambulators, while K3 is for those who can perform activities requiring more advanced prosthetic use. K4 is assigned to individuals with the ability for prosthetic ambulation during activities with high-energy demands [54].

Assessment and classification according to K-levels were conducted by two experienced physiotherapists who were unaware of the study goals.

In addition, the Timed Up and Go Test (TUG) and the Two-Minute Walk Test (TMWT) were performed at the end of the inpatient rehabilitation treatment. These tests were conducted by the same examiners who evaluated the K-levels.

The TUG test was conducted first, according to the previously established protocol for persons with LLL [55]. At the beginning of the test, the participant sat on a standard chair with an armrest (seat height 47 cm, armrest height 74 cm), leaning their back on the back of the chair, with their hands on the armrests, and a gait aid they use in their hand. Participants walked in their shoes and used the aids they used during the rehabilitation treatment. After being seated in the chair, the participant was given instructions for the task, and a distance of 3 m from the chair was marked with white tape. Upon confirming readiness, indicated by the participant, a stopwatch was initiated upon the “go” signal. The participant rose from the chair, proceeded to the marked spot on the floor, turned, walked back to the chair, and resumed a seated position. The test concluded when the participant’s buttocks made contact with the chair seat, at which point the timing was stopped. Participants ambulated at their self-selected pace, and the time was recorded in seconds [55,56].

The TMWT was performed next, according to the modified version of previously designed protocol for persons with LLL [57]. For safety reasons, participants rested for a few minutes after the previous test. Once they confirmed their readiness, they moved to the L-shaped corridor with a longer arm of 35 m and a shorter arm of 15 m. Participants were given instructions on the directions and were advised to walk at a speed that suited them best (self-selected speed). The test began at the start of the corridor. If the participant reached the end of the L-shaped path (50 m), they made a quick turn and returned to the starting point of the corridor, repeating this process. All instructions were provided before the test began, and, if necessary, a physiotherapist was present during the test in addition to the researcher conducting it. The test started in a standing position, with the “go” signal indicating the start of the stopwatch. After two minutes, the distance covered by the participant was recorded, and the total distance was measured in meters [56,57].

### 2.3. Data Preprocessing and Features Extraction

**Figure 1:**
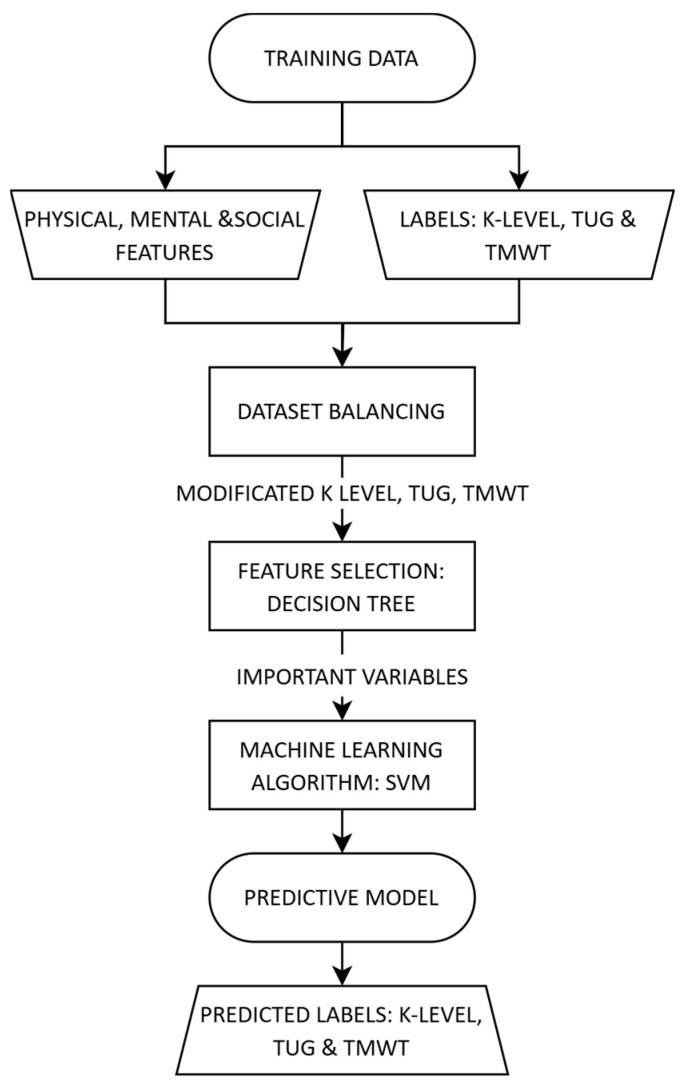
Flowchart of the three-step classification process.

Classification models in medicine usually have two primary objectives. Firstly, they should be able to identify possible nonlinearities that are typical for such complex systems while maintaining accuracy. Secondly, they should be explainable or interpretable, or at least provide insight into logical connections between the inputs and outputs of the model. It is obvious that there is a trade-off between accuracy and understanding of the classification model. To address this issue, we have designed a three-step classification formalism that balances the main goals of accuracy and interpretability (see Figure 1).

#### 2.3.1. Data Balancing

**Figure 2:**
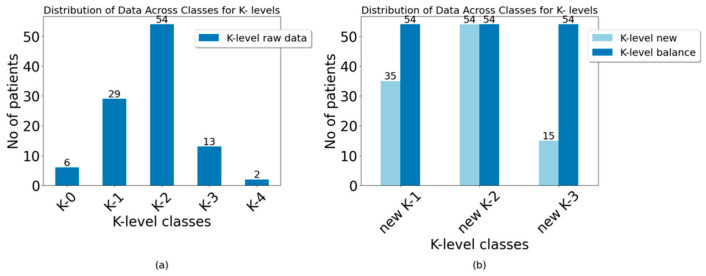
K-levels’ classes bar chart: (**a**) raw data; (**b**) after recategorization and balancing.

The dataset was initially assessed for balance. The primary outcome, determined based on domain knowledge and the researched literature [44,58,59], was the K-level. The histogram below (Figure 2a) illustrates the number of participants/samples at each K-level.

A significant difference was found between the number of participants at each level. Machine learning classifiers fail to cope with imbalanced datasets because these algorithms tend to favor the majority class (the class with the largest number of samples), which can result in false accuracy [58]. To address this issue and ensure the model’s robustness, a modification was made to the classes. A strategic class modification was implemented to mitigate the imbalance and improve the model performance (Figure 2) based on established methodologies [44]. Specifically, classes K0 and K1 were merged into a single category, now referred to as new K1. Class K2 was renamed new K2, while classes K3 and K4 were combined into another category, new K3. This adjustment aimed to create a more balanced representation across modified K-levels, facilitating more accurate predictions in the subsequent model development stages. By modifying the classes, the initial disparity in participant numbers among classes was partially alleviated. However, some imbalances persisted, which could still lead to biases favoring the majority class during model training. Additional steps were taken to further mitigate this issue by randomly duplicating samples from the minority class (Figure 2b).

In addition to predicting K-levels, this study also aimed to assess outcomes related to the TUG test and TMWT, which are numerical assessments. Due to the constraints of a small dataset, and to enhance the model’s predictive performance, these numerical parameters were transformed into categorical types using the following approach:

Participants’ performance times in the TUG test were categorized into predefined intervals based on clinical relevance and the prior literature for a 3 m walking distance, as follows: class TUG1: >60 s; class TUG2: 30–60 s; and class TUG3: <30 s.

Similarly, results from the TMWT test, which measures walking distance over a specific time of 2 min, were categorized into the following relevant intervals: class TMWT1: <25 m; class TMWT2: 25–55 m; and class TMWT3: >55 m.

**Figure 3:**
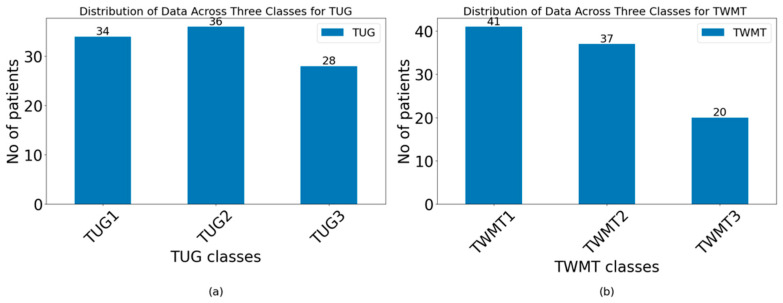
Classes bar chart: (**a**) TUG; (**b**) TMWT.

To visually represent the distribution of participants across the categories derived from the TUG test and TMWT, bar charts were generated, and they are shown in Figure 3.

#### 2.3.2. Feature Selection

Due to the high number of features and the small number of samples (*n* = 104) in the dataset, designing a prediction model using all available features is challenging [60]. Including all features introduces a trade-off regarding model simplicity, experimental design, and output data quality. Additionally, collecting all of the features is a time-consuming process. Therefore, the second step was to reduce the number of input features.

**Figure 4:**
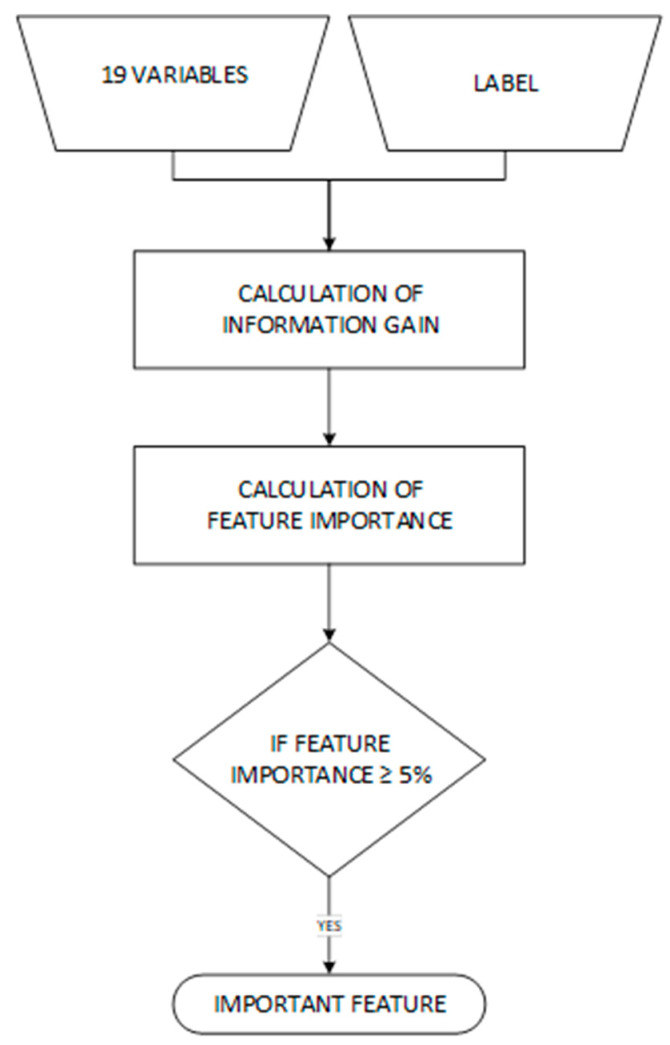
Flowchart of feature selection process.

Feature selection was performed using a decision tree model (Figure 4). This approach allowed us to identify the most relevant features by evaluating their importance in making accurate predictions. Decision trees are particularly effective for feature selection because they can handle numerical and categorical data and provide clear insights into the relationships between features and outcomes. The principle of using decision trees for feature selection, as outlined in references [40,61], involves evaluating feature importance based on how well each feature contributes to reducing prediction uncertainty. Decision trees assess the significance of each feature by measuring how much each feature contributes to the information gain or reduction in entropy within the model. Features that lead to more significant reductions in impurity or entropy are considered more important. Additionally, the depth of the tree reflects feature importance, wherein features appearing closer to the root of the tree are deemed more critical. We limited the tree depth to focus on these key features, ensuring that only the most impactful ones were selected.

Information gain, calculated based on entropy (a measure of uncertainty), was used to assess feature importance [40,61]. The decision tree classifier was chosen because it is straightforward to implement and is widely used in medical dataset analysis [37,38,41]. Its simplicity also makes it easy to interpret, providing a clear understanding of how individual features impact the outcomes. This interpretability is crucial in medical diagnostics, as it offers insight into the significance of each feature in predicting results.

To reiterate, feature selection and dimensionality reduction were essential steps in mitigating the risk of overfitting, which is common when using a large number of input variables. These techniques also help to improve model sensitivity. By reducing the feature set to focus on the most relevant variables, we enhanced the model’s robustness and interpretability, while simultaneously increasing its clinical applicability.

Therefore, a reduced number of features were selected using the decision tree algorithm, which was subsequently used as input for a more complex machine learning procedure.

### 2.4. Mathematical MODEL for Prediction

Based on previous experience [44], and because of the small dataset, the SVM classifier [32,33,34,35,36,62] was designed. The basic idea of an SVM classifier is to classify each sample from the input dataset into one of the two possible classes. Principally, SVM looks for a function of the hyperplane H, which has a role in separating margins for the following two states (classes) of the system: H: ω^T^ x + b = 0, as well as the following two hyperplanes: H1: ω^T^ x + b = 1 and H2: ω^T^ x + b = −1, if there are no points/samples between H1 and H2, and if the distance is maximal. The weights, ω, and constant, b, are determined through the learning process. If the surface separating the two classes is nonlinear, a kernel function is introduced. The function transforms the input data from a low-dimensional space to a high-dimensional space, in which the surface between the classes is linearly separable. The choice of the kernel function is completed experimentally, with the optimization criteria being the accuracy of classification. Accuracy is defined as the number of correct predictions in all predictions made. The SVM algorithm can also be used for multiclass problems. One approach is the one vs. one method, which forms C(C − 1)/2 classifiers (one for each pair of classes), where C is the number of classes.

## 3. Results

A total of 104 participants, described by nineteen variables and three tests (K-level, TUG, TMWT), including 80 (76.9%) men and 24 (23.1%) women, with an average age of 62.1 ± 10.9 (19–82) years, were enrolled in the study. Six patients were not able to complete the TUG and TMWT at follow-up and were excluded from the prediction model.

Characteristics of the participant sample (*n* = 104) are shown in Table 1.

In this work, an SVM approach paired with decision tree feature elimination was utilized to build predictive models for the K-level, TUG test, and TMWT. A previously described decision tree method was employed to select features that optimally distinguish the level of ability to walk with a prosthesis after lower limb amputation.

**Figure 5:**
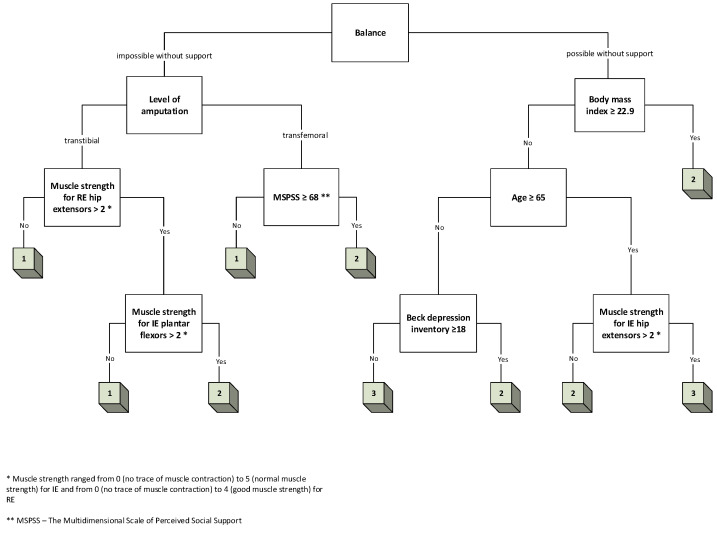
A 4-layer decision tree for K-level.

**Figure 6:**
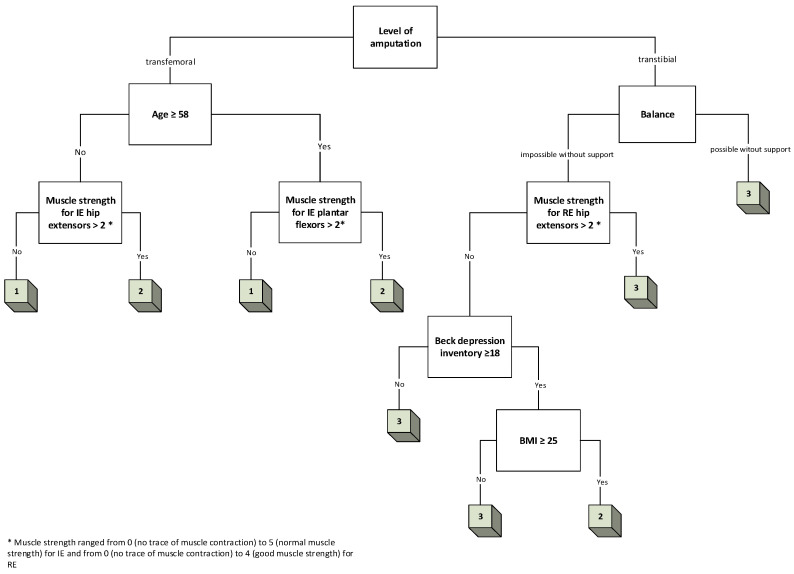
A 5-layer decision tree for TUG.

**Figure 7:**
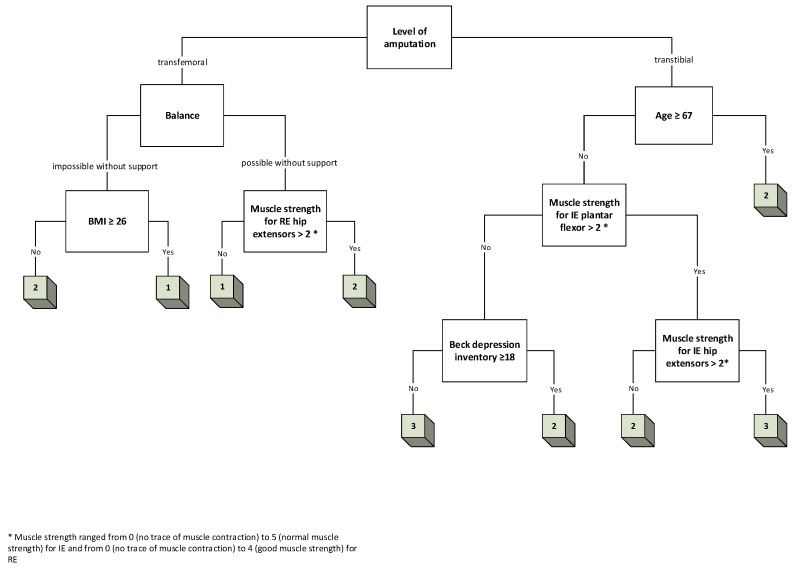
A 4-layer decision tree for TMWT.

Three decision trees were created, one for each of the following outputs: K-level, TUG, and TMWT (Figure 5, Figure 6 and Figure 7, respectively).

For the K-level, a decision tree with four layers and nine different variables was constructed, identifying the nine key variables associated with different levels of walking ability in individuals with LLL. This model selected the following features: balance, BMI, age, BDI, MSPSS, amputation level, muscle strength of the residual extremity (RE) hip extensors, intact extremity (IE) plantar flexors, and IE hip extensors

Similar procedures were followed to create decision trees for the TUG and TMWT tests. The decision trees for these outcomes were constructed based on the same principle of feature selection using information gain, and the same features were selected for both models, excluding MSPSS (eight features).

The next step involved data balancing, where the number of samples for the K-level prediction model was artificially increased to 162. This procedure was implemented to address initial class imbalances in the dataset, ensuring a more equitable representation of different K-level categories. Predictive models were developed using support vector machines (SVM) with various kernel functions to predict the K-level, TUG test, and TMWT outcomes in individuals with LLL. The models were evaluated based on their accuracy, sensitivity, and precision. The results are shown in Table 2.

The input features for these models were previously selected through feature selection by using a decision tree with information gain. To evaluate the generalization and performance of the model, the dataset was divided into training and validation sets, utilizing 10-fold cross-validation.

SVM predictive models, using a radial basis function (RBF) kernel, were developed to predict the K-level, TUG, and TMWT outcomes in participants with LLL. These models used nine (or eight) input variables. For the K-level, the SVM model achieved an impressive accuracy of approximately 93%, correctly classifying 141 out of 162 samples. It is crucial to highlight that the number of samples exceeds the number of actual participants due to a data balancing procedure, implemented to address class imbalance, where artificial data were added. The models for the TUG and TMWT outcomes both achieved approximately 83% accuracy.

**Figure 8:**
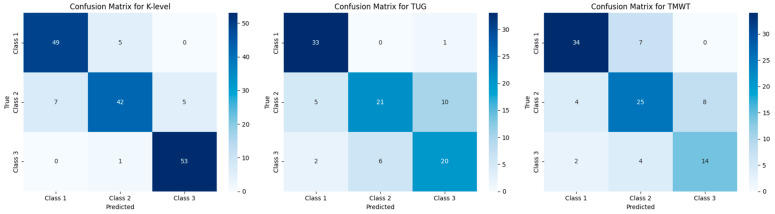
Confusion matrix for the SVM model’s outcomes.

The detailed results, including all performance indicators, are shown in Table 3 and Figure 8.

For the results of additional numerical experiments, see Appendix A.

## 4. Discussion

### 4.1. Key Predictors of Functional Outcomes

The primary motivation for applying prediction methods in medicine is to reduce the risk of incorrect diagnoses or treatments. Accurate prediction of walking ability after lower limb amputation plays a critical role in patient rehabilitation and forms the basis for appropriate prosthesis prescription [44]. Traditionally, a physicians’ decision has been reliant on their experience and expertise, but machine learning systems could be the ones that would help physicians with a “second opinion”. The integration of machine learning techniques into medical practice has gained significant momentum in recent years, particularly in the realm of predictive modeling in diagnosis and prognosis across various medical conditions [59]. This paper aims to contribute to this growing body of research by employing machine learning methods to develop a prediction model for assessing the level of walking ability with a prosthesis following lower limb amputation, as well as the TMWT level and TUG class, as described in the methodology part of this paper. Our study focused on leveraging support vector machines (SVMs) in conjunction with decision tree-selected input variables to construct predictive models to predict functional outcomes and the K-level of mobility.

Out of the nineteen prediction variables we began with, our prediction SVM models (with radial basis function) created prediction models with nine features for the K-level and eight features for the TUG and TMWT. The following eight prediction parameters are mutual for all three outcomes: balance, age, level of amputation, BMI, BDI, muscle strength of IE hip extensors and plantar flexors, and RE hip extensors. MPSS was an important part only of the decision tree for the K-level. For the K-level, balance was the most significant, and it helped exclude either the highest or the lowest level. The level of amputation plays a similar role in determining the TMWT level and TUG class, with a transfemoral level of amputation directing the decision tree to less favorable levels and classes. The above-mentioned predictors are well-known and have been established in previous studies [21,44].

Upon closer examination of the predictive factors identified by our decision tree analysis, several key determinants emerge as pivotal contributors to the accuracy of our prediction models. For K-level classification, balance, body mass index (BMI), and level of amputation emerged as the most influential predictors. This aligns with the existing literature highlighting the significance of these factors in determining functional outcomes following lower limb amputation [63]. Higher BMI in individuals with LLL has been associated with decreased functional outcomes [64], including slower walking speed and reduced endurance. Excess weight can exacerbate biomechanical challenges and increase energy expenditure during walking with a prosthesis. In contrast to the study conducted by Linberg et al. [65], where BMI did not significantly influence the functional outcome, our prediction model found that BMI is a strong predictor of the K-level, with the cut-off value being 22.9, with higher values negatively impacting the outcome [64].

Similarly, our analysis revealed age, balance, and level of amputation as primary predictors for both TUG and TMWT classifications. Notably, older age was associated with lower TUG and TMWT scores, underscoring the impact of age-related physiological changes on functional mobility post-amputation [60]. Balance emerged as a consistent predictor across all three functional assessments, emphasizing its critical role in post-amputation rehabilitation outcomes. Balance is essential for safe and efficient mobility, especially for individuals with lower limb amputations who rely on prostheses [66]. Poor balance control can lead to an increased risk of falls and reduced confidence in performing daily activities. In a study by Sions et al., it was found that impaired balance has been associated with decreased performance on functional tests, such as the TUG test and the six-minute walk test (SMWT), among individuals with LLL [67].

Our model also points to the predictive role of balance, with differences between the previously mentioned study and our research being that we performed balance tests instead of using self-report balance assessment, and instead of SMWT, we used TMWT. According to our model, the ability to maintain balance without support favors a higher level of mobility and functionality. Regarding this parameter, a lot of support already exists in most of the previous findings [13,18,68]. The level of amputation plays a similar role in determining TMWT level and TUG class, with the transfemoral level of amputation directing the decision tree to less favorable levels or classes, a finding that is in line with already established estimations [13,65,69,70].

### 4.2. Psychological and Social Factors

Furthermore, our decision tree analysis highlighted the relevance of psychological and social factors, such as depression and perceived social support, in predicting the K-level. Our model used BDI as a predictive factor. Previous studies found a strong correlation between this inventory and the functional outcome [9,71]. According to Desmond and MacLachlan, amputations significantly alter the patient’s day-to-day activities, particularly in terms of psychosocial interactions [72]. A physical impairment can result in feelings of hopelessness, melancholy, anxiety, low self-esteem, stigma, loneliness, and weakness [73,74]. Higher levels of depression have been associated with poorer performance during functional tests among individuals with LLL [73]. Social support plays a significant role in coping with limb loss and adjusting to life with a prosthesis [75]. Our model indicates that higher levels of perceived social support have been associated with higher levels of mobility, with the cut-off value being 68 (higher values favor a better level of mobility). This connection could be explained by better psychological well-being, which can indirectly influence functional outcomes through improved motivation and self-efficacy. This underscores the importance of addressing psychosocial factors in comprehensive rehabilitation programs for individuals with lower limb amputations [9,20].

Our model identified muscle strength as a key predictor, which aligns with established research on functional outcomes in individuals with lower limb amputation. Stronger muscles, particularly in the hip extensors and plantar flexors of the intact extremity, as well as the hip extensors of the residual limb, have consistently been associated with higher K-level classifications [76,77,78]. This correlation suggests that greater muscle strength enhances functional capacity and overall mobility with a prosthesis. In addition to its impact on K-level classification, enhanced muscle strength in both intact and remaining extremities has been shown to positively influence performance on functional mobility tests such as the TMWT and TUG tests [79]. Individuals with greater muscle strength tend to exhibit improved endurance and agility, leading to better performances on these standardized assessments [80]. The positive correlation between muscle strength and functional outcomes underscores the importance of targeted strength training interventions as part of comprehensive rehabilitation programs for individuals with LLL [81]. Furthermore, including muscle strength as a predictor in our SVM models reinforces its significance in predicting the K-level, TMWT, and TUG classifications. By incorporating objective measures of muscle strength into predictive models, clinicians can better tailor rehabilitation interventions to address specific deficits and optimize functional outcomes for individuals undergoing prosthetic rehabilitation following lower limb amputation.

### 4.3. Advantages of Machine Learning in Predictive Modeling

While several predictors identified in our study have been established in previous research [14,15,16], the use of ML offers significant advantages. Traditional prediction models often focus on a limited number of variables and may not fully capture the complex interactions between different factors [44]. In contrast, ML models can simultaneously analyze larger datasets, encompassing a wide range of clinical parameters, and leading to more precise and individualized predictions [82]. Furthermore, our model demonstrated high accuracy when tested on an independent sample, highlighting its potential for broader application. This suggests that ML could uncover more efficient predictors that might be missed by using traditional methods, thereby improving clinical decision-making and patient outcomes.

Our study differs from the work of Quek et al. (2024) in our selection of input features, focusing more on muscle strength and functional tests rather than demographic factors [26]. However, both studies demonstrate the potential of machine learning to enhance prosthetic prescription and mobility assessment. Nsugbe et al. (2024) further emphasize the role of wearable technology, which could complement traditional assessments such as TUG and TMWT in future models to achieve real-time mobility tracking [27].

The clinical implications of our study are significant, as they provide a robust framework for assessing functional outcomes in individuals with LLL. By integrating these models into routine clinical practice, clinicians can make more relevant decisions regarding prosthesis prescriptions for individuals with LLL. The ability to accurately predict thr K-level, TUG class, and TMWT class can facilitate early interventions and ultimately improve the quality of life for patients. Moreover, these models offer a valuable second opinion tool for clinicians, complementing their expertise and helping to mitigate the risks of subjective bias in clinical decision-making [59].

### 4.4. Strengths and Limitations

The primary strength of the present study is that we covered physical, psychological, and social factors that could potentially influence rehabilitation outcomes. However, there are several limitations that we need to address. The first limitation of this study is the relatively small sample size and single-site data collection. This constraint limits the generalizability of the findings and the robustness of the predictive models. Although our dataset consisted of 104 patients, the imbalanced distribution across K-levels (K0: 6, K1: 29, K2: 54, K3: 13, K4: 2) may have influenced the results, leading to potential overfitting. To mitigate this, we applied data balancing techniques and performed a statistical power analysis, which revealed a power value close to 1, indicating a high probability of detecting significant differences, should they exist. Additionally, we calculated Cohen’s ω [83], yielding a large effect size of 0.9129, thus reinforcing the reliability of our findings. SVMs are generally robust for smaller datasets, compared to other algorithms, because they can perform well even with a limited number of samples [62]. The issue of an unbalanced dataset also persists, as models tend to favor the majority class, potentially skewing results. Although data balancing techniques were applied, this can sometimes create an artificial picture that does not accurately reflect real-world conditions. We also acknowledge a potential risk of bias in our study due to the broad inclusion criteria, particularly concerning the different amputation levels. While the rehabilitation program was individualized, the variations in the level of amputation could have influenced the outcomes, and this should be considered when interpreting the results. The next limitation of this study is the lack of follow-up of these patients. Therefore, we are not aware of whether these predictors will still be significant in predicting the functional level in the future. For future research, we suggest using bigger datasets, as well as follow-up evaluations of the functional status of these patients. By having a larger and more balanced dataset, the ML model can generalize better to unseen data. This reduces the risk of overfitting and ensures that the model’s predictions are more reliable in real-world scenarios [84,85]. To validate our model further, we employed a 10-fold cross-validation approach, achieving an average accuracy of 92.59%, with a standard deviation of 0.21%. These results demonstrate the robustness and reliability of the model’s performance. With more samples in each class and test algorithm over the original K-levels, we would be able to develop software for K-level prediction, which could be used in everyday clinical practice. It should also be mentioned that balance testing was only performed on the intact leg, as testing on the prosthetic side was not part of the protocol when the data were collected. However, most researchers test balance on the intact leg because, at the beginning of prosthetic fitting and training, individuals with lower limb loss have not learned to use their prosthesis yet, making these tests unrealistic [56].

Future studies should consider including balance assessments—after prosthetic fitting and training—on the prosthetic side, to provide a more comprehensive evaluation of the participants’ functional abilities. A potential limitation of our study could be an L-shaped corridor where TMWT was performed, as volunteers needed to complete a 90-degree turn after 35 m and a 180-degree turn after 50 m. However, we believe that this limitation did not significantly influence the results obtained during TMWT, since, in previous studies, TMWT was performed on even shorter corridors [86]. Lastly, our study sample primarily comprised individuals with limb loss due to dysvascular conditions, which is associated with different rehabilitation outcomes compared to traumatic amputations [87]. This homogeneity may limit the applicability of our findings to other groups, such as those with traumatic limb loss. Furthermore, the sample was predominantly male, which may affect the generalizability of our results to the entire population, particularly to female patients.

## 5. Conclusions

In conclusion, by using an SVM model with nine variables (balance, BMI, age, BDI, MSPSS, amputation level, muscle strength of the residual extremity (RE) hip extensors, IE plantar flexors, and IE hip extensors), the K-level, TUG class, and TMWT level can be predicted with high accuracy. These clinical assessments could be incorporated into routine clinical practice to guide clinicians and inform patients of their potential level of ambulation.

## Figures and Tables

**Table 1:** Demographic and clinical characteristics of participants.

Variable		Value
Average age in years ± SD (min–max)		62.1 ± 10.9 (19–82)
Gender	Men *n* (%)	80 (76.9)
	Women *n* (%)	24 (23.1)
BMI ^1^ (kg/m^2^) (Mean ± SD (min–max))		24.8 ± 3.7 (16.5–36.2)
Number of years of education (Mean ± SD (min–max))		10.15 ± 3.38 (0–18)
Tobacco smoking	Non-smoker	38 (36.5)
	Smoker	34 (32.7)
	Ex-smoker ^2^	31 (29.8)
Diabetes mellitus	Yes *n* (%)	74 (71.2)
	No *n* (%)	30 (28.8)
FCI ^3^ (Mean ± SD (min–max))		2.91 ± 1.53 (0–8)
Cause of amputation	Dysvascular *n* (%)	92 (88.5)
	Traumatic *n* (%)	7 (6.7)
	Other causes *n* (%)	5 (4.8)
Level of amputation	Transtibial *n* (%)	40 (38.5)
	Transfemoral *n* (%)	64 (61.5)
Phantom limb pain	Yes *n* (%)	69 (68.3)
	No *n* (%)	32 (31.7)
Contracture	Yes *n* (%)	26 (25.0)
	No *n* (%)	78 (75.0)
Balance	0 *n* (%)	2 (1.9)
	1	22 (21.2)
	2	33 (31.7)
	3	47 (45.2)
RE ^4^ hip extensors strength (Mean ± SD (min–max))		3.12 ± 0.66 (2–4)
IE ^5^ plantar flexors strength (Mean ± SD (min–max))		3.34 ± 1.09 (1–5)
IE ^5^ hip extensors strength (Mean ± SD (min–max))		3.85 ± 0.79 (2–5)
Time period since amputation till prosthetic fitting (days) (Mean ± SD (min–max))		172.0 ± 98.0 (43–517)
MSPSS ^6^ (Mean ± SD (min–max)		5.9 ± 1.2 (1.5–7.0)
MMSE ^7^ (Mean ± SD (min–max))		26.55 ± 2.98 (16–30)
BDI ^8^ (Mean ± SD (min–max))		11.43 ± 8.10 (0–35)
K-level	K0 *n* (%)	6 (5.76)
	K1 *n* (%)	29 (27.88)
	K2 *n* (%)	54 (51.93)
	K3 *n* (%)	13 (12.5)
	K4 *n* (%)	2 (1.9)
TUG ^9^ (s) (Mean ± SD (min–max))		35.89 ± 23.61 (4–105)
TMWT ^10^ (m) (Mean ± SD (min–max))		63.18 ± 43.16 (14–221)

^1^ Body mass index, ^2^ persons who quit smoking in the previous 6 months, ^3^ Functional Comorbidity Index, ^4^ residual lower extremity, ^5^ intact lower extremity, ^6^ Multidimensional Scale of Perceived Social Support, ^7^ Mini Mental State Exam, ^8^ Beck depression inventory, ^9^ Timed Up and Go, ^10^ Two-minute walking test.

**Table 2:** Accuracy (%) of different kernel functions for K-level, TUG, and TMWT.

Type of Kernel Function	K-Level Accuracy (%)	TUG ^1^ Accuracy (%)	TMWT ^2^ Accuracy (%)
Radial Basis Function	92.59	83.67	82.99
Linear	79.48	74.15	74.23
Polynomial	87.64	80.23	79.99

^1^ Timed Up and Go, ^2^ two-minute walking test.

**Table 3:** Indicators of successful classification and prediction.

Metric	K-Level	TUG ^1^	TMWT ^2^
Sensitivity (%)	88.89	75.6	73.49
Specificity (%)	94.44	87.89	87.06
Accuracy (%)	92.59	83.67	82.99
Precision (%)	88.79	74.93	72.69

^1^ Timed Up and Go; ^2^ two-minute walking test.

## Data Availability

The dataset and analysis scripts used in this study are available at https://github.com/mirnak88/WalkingAbility.

## Appendix A. Comparative Analysis of Machine Learning Classifiers

To provide context for the selection of the SVM model, we also evaluated the following two other machine learning classifiers commonly applied in small datasets: k-Nearest Neighbors (k-NN) and Naive Bayes. Each model was tested using the same set of input features for predicting the K-level. Results indicated that k-NN achieved an accuracy of 80.97%, Naive Bayes achieved 89.64%, and SVM achieved the highest accuracy, at 92.59%. Based on these findings, SVM was chosen as the primary model due to its superior performance, particularly in managing nonlinear data, and in its robustness with smaller datasets.
